# CDK4/6 inhibitors in drug-induced liver injury: a pharmacovigilance study of the FAERS database and analysis of the drug–gene interaction network

**DOI:** 10.3389/fphar.2024.1378090

**Published:** 2024-04-03

**Authors:** Youjun She, Zihan Guo, Qing Zhai, Jiyong Liu, Qiong Du, Zhongwei Zhang

**Affiliations:** ^1^ Department of Pharmacy, Fudan University Shanghai Cancer Center, Shanghai, China; ^2^ Department of Oncology, Shanghai Medical College, Fudan University, Shanghai, China; ^3^ Department of Critical Care, Fudan University Shanghai Cancer Center, Shanghai, China

**Keywords:** cyclin-dependent kinase 4/6 inhibitors, drug-induced liver injuries, pharmacovigilance, FAERS, disproportionality analyses, protein-protein interaction

## Abstract

**Objective:**

The aim of this study was to investigate the potential risk of drug-induced liver injury (DILI) caused by the CDK4/6 inhibitors (CDK4/6is abemaciclib, ribociclib, and palbociclib by comprehensively analyzing the FDA Adverse Event Reporting System (FAERS) database. Moreover, potential toxicological mechanisms of CDK4/6is-related liver injury were explored via drug–gene network analysis.

**Methods:**

In this retrospective observational study, we collected reports of DILI associated with CDK4/6i use from the FAERS dated January 2014 to March 2023. We conducted disproportionality analyses using the reporting odds ratio (ROR) with a 95% confidence interval (CI). Pathway enrichment analysis and drug-gene network analyses were subsequently performed to determine the potential mechanisms underlying CDK4/6i-induced liver injury.

**Results:**

We found positive signals for DILI with ribociclib (ROR = 2.60) and abemaciclib (ROR = 2.37). DILIs associated with liver-related investigations, signs, and symptoms were confirmed in all three reports of CDK4/6is. Moreover, ascites was identified as an unlisted hepatic adverse effect of palbociclib. We isolated 189 interactive target genes linking CDK4/6 inhibitors to hepatic injury. Several key genes, such as STAT3, HSP90AA1, and EP300, were revealed via protein-protein analysis, emphasizing their central roles within the network. KEGG pathway enrichment of these genes highlighted multiple pathways.

**Conclusion:**

Our study revealed variations in hepatobiliary toxicity among the different CDK4/6 inhibitors, with ribociclib showing the highest risk of liver injury, followed by abemaciclib, while palbociclib appeared relatively safe. Our findings emphasize the need for cautious use of CDK4/6 inhibitors, and regular liver function monitoring is recommended for long-term CDK4/6 inhibitor use.

## 1 Introduction

Cyclin-dependent kinase 4/6 inhibitors (CDK4/6is), such as palbociclib, ribociclib, and abemaciclib, have been approved for treating patients with hormone receptor-positive and human epidermal growth factor receptor 2-negative breast cancer (Finn et al., 2015; Cristofanilli et al., 2016; Finn et al., 2016; Hortobagyi et al., 2016; Dickler et al., 2017; Goetz et al., 2017; Sledge et al., 2017; Slamon et al., 2018; Tripathy et al., 2018; Turner et al., 2018; Johnston et al., 2020; Royce et al., 2022). With a median progression-free survival (PFS) exceeding 2 years in first-line metastatic patients, indicating long-term use, evaluating the enduring safety of CDK4/6is in breast cancer treatment is imperative (Gao et al., 2020; Harbeck et al., 2021).

While these drugs exhibit similar clinical efficacy, their adverse event (AE) spectra differ markedly (Asghar et al., 2015; Desnoyers et al., 2020; George et al., 2021). To assess the safety of CDK4/6is, it is essential to evaluate their risk for rare adverse effects, such as drug-induced liver injuries (DILIs), which can range from mild test result abnormalities to severe liver failure (David and Hamilton, 2010; Bøttcher et al., 2019; Desnoyers et al., 2020). Despite the low incidence of DILI, the severity of this disease is concerning. Current adverse drug reaction (ADR) data for CDK4/6is are predominantly from short-term clinical trials and cohort studies and may not capture rare DILI events (Bøttcher et al., 2019; Desnoyers et al., 2020). Therefore, collecting additional data from real-world settings and extending the follow-up duration are necessary to accurately measure DILI risk.

Spontaneous adverse event reporting, a valuable source of real-world evidence, is facilitated by databases such as the Food and Drug Administration Adverse Event Reporting System (FAERS) (Goldman, 1998; Toki and Ono, 2018). Disproportionality methods are often used to automatically obtain signals about drug safety from large databases (Montastruc et al., 2011). To determine whether DILI is associated with CDK4/6is, we analyzed the FAERS database using disproportionality analysis. To inform clinical practice, we compared signals for hepatic injuries caused by different CDK4/6is.

The exploration of drug‒gene interactions has advanced our understanding of drug toxicity (Hahn and Roll, 2021). Recent studies have proposed combined analyses using FAERS and drug–gene interaction data to enhance our knowledge of adverse events (AEs) (Tanaka et al., 2021). However, the mechanisms underlying CDK4/6i-induced liver injury are unclear. To address this gap, we constructed a drug‒gene interaction network utilizing datasets of human genes interacting with CDK4/6 inhibitors and genes associated with liver injury. Functional enrichment analyses were subsequently applied to determine the potential toxicological mechanisms of CDK4/6 inhibitor-associated liver injury.

## 2 Materials and methods

### 2.1 FAERS data extraction and mining

We executed a retrospective observational pharmacovigilance study using OpenVigil 2.1-MedDRA (http://openvigil.sourceforge.net), a publicly available tool for pharmacovigilance analysis on the FAERS database that does not require any special licenses or statistical programs (Böhm et al., 2016). Our study collected adverse reaction data from January 2014 to March 2023 and categorized the patients according to the Medical Dictionary for Regulatory Activities (MedDRA) classification system. We analyzed preferred terminology (PT), high-level terminology (HLT), and standardized MedDRA queries (SMQs) to comprehensively identify and classify ADRs (Pearson et al., 2009; Vogel et al., 2020; MedDRA, 2022).

To improve signal detection, we applied eight SMQs (as shown in Table 1) in the “Drug-related hepatic disorders - comprehensive search” and 324 PTs at lower SMQs to classify adverse events related to liver disorders.

**TABLE 1:** Standardized MedDRA query (SMQ) terms for performing liver injury signal evaluation.

Code	SMQ terms
20000008	Liver related investigations, signs and symptoms (SMQ)
20000013	Hepatic failure, fibrosis and cirrhosis and other liver damage-related conditions (SMQ)
20000009	Liver tumors of unspecified malignancy (SMQ)
20000010	Hepatitis, noninfectious (SMQ)
20000209	Liver tumors of unspecified malignancy (SMQ)
20000208	Liver malignant tumors (SMQ)
20000015	Liver-related coagulation and bleeding disturbances (SMQ)
20000012	Liver neoplasms, benign (incl cysts and polyps) (SMQ)

### 2.2 Disproportionality analysis and signal detection

Disproportionality analysis is a statistical method used in pharmacovigilance to identify possible AEs (Montastruc et al., 2011). For this study, it compares the frequency of reporting of a specific liver-related AE associated with a CDK4/6 inhibitor with the frequency of that event for all other drugs in the database. To determine whether CDK4/6 inhibitors have a higher-than-expected rate of reported adverse events, statistical metrics such as the reporting odds ratio (ROR) were calculated, indicating a potential safety signal (Bate and Evans, 2009).

**FIGURE 1:**
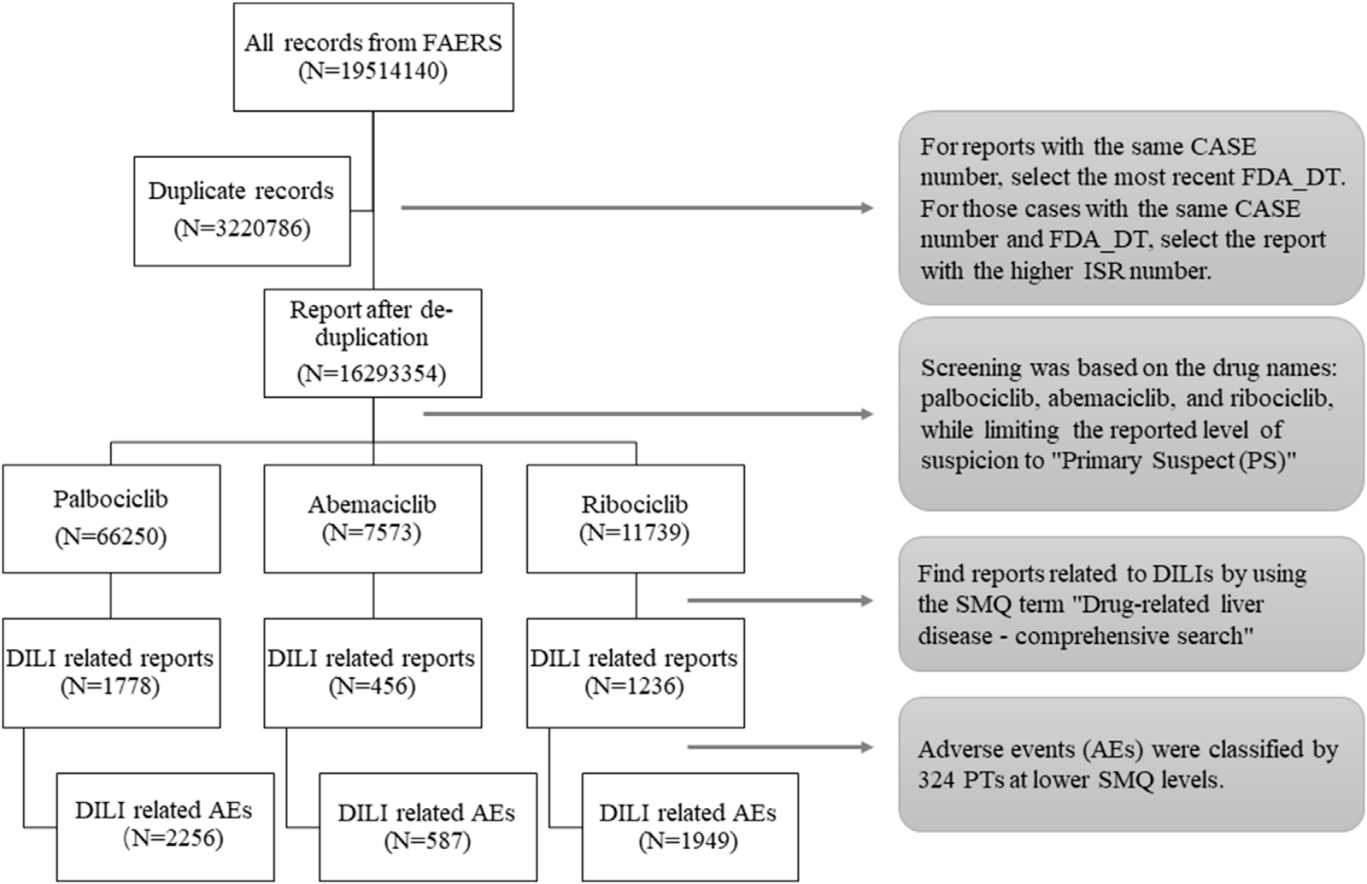
Flow chart of data extraction. A detailed description of the data extraction process for drug-induced liver injury (DILI) adverse events for CDK4/6 inhibitors in the U.S. Food and Drug Administration Adverse Event Reporting System (FAERS).

The analysis focused on reports that were marked as “major suspicious” for the drugs “palbociclib,” “ribociclib,” and “abemaciclib” in the FAERS database. To ensure accuracy, duplicate reports were removed (as shown in Figure 1). The ROR method was applied using OpenVigil 2.1-MedDRA-v24. To identify liver-related AE signals associated with CDK4/6 inhibitors compared to other drugs in the FAERS database. The criteria for positive AE signals included at least three AE reports and a lower limit of the 95% confidence interval (CI) of the ROR greater than 1 to minimize false positive signals (Rothman et al., 2004; Bate and Evans, 2009; Montastruc et al., 2011).

### 2.3 Network analysis of CDK4/6is-hepatic injury gene interactions

Network analysis is an interdisciplinary approach that delves into the interactions between drugs and biological systems at the network level. It integrates various types of biological data, including drug-target interactions, protein‒protein interactions, gene expression profiles, and disease associations, into comprehensive network models (see Table 2 for definitions). In this study, biological entities such as CDK4/6is, targets, genes, and proteins associated with liver injury are depicted as nodes in the network, while their interactions are represented as edges. By employing graph theory and network analysis techniques to scrutinize the properties of these networks, we aimed to predict the potential targets and pathways involved in liver injury induced by CDK4/6is.

**TABLE 2:** Definition of pharmacovigilance and pharmacogenetic terms.

Term	Defination
FAERS	FDA Adverse Event Reporting System, a database maintained by the U.S. Food and Drug Administration (FDA) that contains reports of medication errors and adverse events
ROR	Reporting Odds Ratio, a statistical tool frequently utilized in pharmacovigilance to detect signals in databases of reported adverse events. It measures the degree of correlation between a specific drug and a particular adverse event in comparison to all other drugs present in the database
Drug-gene interactions	Interactions between drugs and specific genetic variants (polymorphisms) that influence drug metabolism, efficacy, or toxicity
Protein-protein interactions (PPI)	Protein-protein interactions occur when two or more proteins physically bind within a biological system. Proteins rarely act independently, but rather participate in complex networks of protein interactions
KEGG pathway analysis	KEGG pathway analysis involves the use of the KEGG database to computationally analyze biological data and identify important biological pathways

#### 2.3.1 CDK4/6is- hepatic injury gene interaction network dataset

We utilized SwissTargetPrediction (http://www.swisstargetprediction.ch) and SuperPred (https://prediction.charite.de) databases to identify genes linked with CDK4/6is (abemaciclib, ribociclib, palbociclib). Genes associated with liver injury were extracted from (https://www.genecards.org) and OMIM (https://www.omim.org) databases using “liver injury” as the keyword.

Gene data underwent curation via the UniProt database. The intersection of drug-associated genes and those related to liver injury formed the basis for constructing the drug-gene network using Cytoscape 3.7.2.

#### 2.3.2 Protein-protein interaction network dataset

Protein-protein interactions were analyzed using the String (https://string-db.org) database focusing on *Homo sapiens* species with a 0.7 interaction score threshold. KEGG pathway analysis through the R package “clusterProfiler (version 1.4.0)” provided insights into biological pathways influenced by gene interactions, visualized using “ggplot2.”

This analysis aims to clarify the interaction between CDK4/6 inhibitors and genes associated with hepatic injury, revealing potential mechanisms underlying drug-induced liver damage.

## 3 Results

### 3.1 Descriptive analysis

A total of 84,462 records associated with CDK4/6is were extracted, revealing 3,470 records (4.1%) linked to DILI AEs. Table 3 outlines patient characteristics relevant to CDK4/6i-induced DILI. The table demonstrates that palbociclib exhibited the highest number of DILI-associated reports, followed by ribociclib and abemaciclib. Notably, hospitalization was the primary outcome among patients affected by DILI. The median onset of DILI occurred approximately 30 days after treatment initiation, with distinct median onset durations observed: 48 days for palbociclib, 32 days for abemaciclib, and 42 days for ribociclib. Intriguingly, during the data deduplication process, 101 patients experienced DILI due to various CDK4/6 inhibitors.

**TABLE 3:** Characteristics of reports on CDK4/6i-associated DILIs in the FAERS database (January 2014 to March 2023).

	Palbociclib	Abemaciclib	Ribociclib
Gender			
Female (%)	1,625 (91.39)	401 (87.94)	1,156 (93.53)
Male (%)	29 (1.63)	4 (0.88)	16 (1.29)
Missing (%)	124 (6.97)	51 (11.18)	64 (5.18)
Age			
N (Missing)	1,475 (303)	273 (183)	710 (526)
Median (q1, q3)	63 (54.70)	62 (54.70)	59 (50.68)
Year of report			
Before 2019 (%)	922 (51.84)	85 (18.64)	290 (23.46)
2020 (%)	223 (12.54)	97 (21.27)	214 (17.31)
2021 (%)	247 (13.89)	95 (20.83)	259 (20.95)
2022 (%)	292 (16.42)	134 (29.39)	340 (27.51)
2023 (%)	94 (5.29)	45 (9.87)	133 (10.76)
Reported by			
Consumers (%)	606 (34.08)	234 (51.32)	505 (40.86)
Health Professionals (%)	1,148 (64.57)	210 (46.05)	719 (58.17)
Unknown (%)	24 (1.35)	12 (2.63)	12 (0.97)
Outcome, n (%)			
Life-Threatening	38 (2.14)	24 (5.26)	86 (6.96)
Hospitalization	430 (24.18)	141 (30.92)	371 (30.02)
Disability	8 (0.45)	6 (1.32)	18 (1.46)
Death	320 (18.00)	55 (12.06)	205 (16.59)
Other	596 (33.52)	139 (30.48)	373 (30.16)
Missing	386 (21.71)	91 (19.96)	183 (14.81)
Time-to-onset, days			
N (Missing)	509 (1,269)	159 (297)	491 (745)
Median (q1, q3)	48 (14.146)	32 (13.63)	42 (14.120)

### 3.2 Signal detection of DILI-related AEs in the FAERS database

Signal detection at the SMQ and PT levels revealed associations between CDK4/6 inhibitors and DILI (as shown in Table 4). A comprehensive search was performed using the SMQ term “Drug-related hepatic disorders.” Abemaciclib (ROR = 2.37) and ribociclib (ROR = 2.60) were shown to be associated with increased incidences of DILI, while palbociclib (ROR = 0.70) did not significantly affect the incidence of DILI.

**TABLE 4:** Disproportionality analyses for CDK4/6i-related DILIs.

CDK4/6 inhibitor	Number of DILIs reports	ROR (95%CI)
Palbociclib	2,256	0.70 (0.67, 0.73)
Abemaciclib	587	2.37 (2.18, 2.58)
Ribociclib	1949	2.60 (2.48, 2.72)

ROR, reporting odds ratio; 95% CI, 95% confidence interval.

**FIGURE 2:**
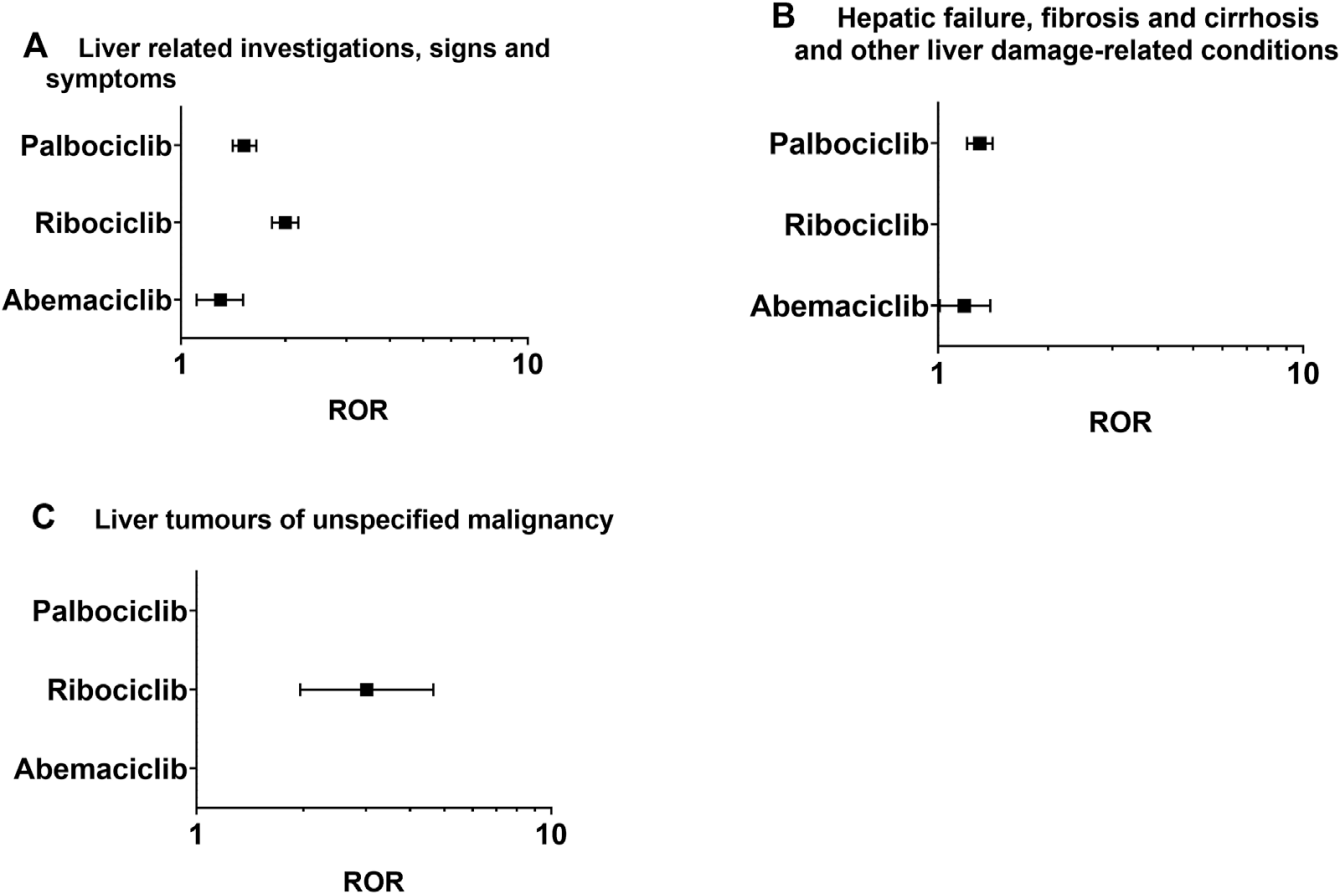
Positive signal distribution for CDK4/6 inhibitors using the standardized MedDRA queries (SMQs). **(A)** Liver-related investigations, signs and symptoms (SMQ); **(B)** hepatic failure, fibrosis and cirrhosis and other liver damage-related conditions (SMQ); **(C)** liver tumors of unspecified malignancy (SMQ). ROR = Reporting odds ratio, statistically positive signals with a lower limit of the 95% confidence interval of ROR greater than 1. Negative signals are not displayed in the figure.

After identifying signals in 8 lower-level SMQ terms (Table 1; Figure 2), all the CDK4/6 inhibitors were found to be associated with liver-related signs and symptoms. Abemaciclib and ribociclib were specifically correlated with hepatic failure, fibrosis, cirrhosis, and other liver damage-related conditions, while ribociclib was associated with unspecified liver tumors.

The results of AE signal detection under PT conditions are shown in Table 5. Ribociclib had positive signals in 36 PT terms, including 196 patients with elevated alanine aminotransferase (ROR = 3.70) and 183 patients with increased aspartate aminotransferase (ROR = 3.99). Abemaciclib had 19 positive signals, primarily related to hepatic function abnormalities (ROR = 8.29). Conversely, palbociclib exhibited seven positive signals, including ascites (ROR = 1.94) in 202 patients and hypertransaminasemia (ROR = 2.58). Moreover, through data mining, several previously unreported adverse events have been discovered that are not mentioned in the CDK4/6 inhibitor labels. These included ascites (N = 323, 6.74%), jaundice (N = 79, 1.65%), hepatomegaly (N = 28, 0.58%), hepatic neoplasm (N = 21, 0.43%), hepatic cytolysis (N = 12, 0.25%), hepatic cirrhosis (N = 11, 0.22%), and hepatic cysts (N = 8, 0.17%).

**TABLE 5:** Positive signal strength for liver injuries associated with CDK4/6is based on PT levels of FAERS.

High-level terminology (HLT)	Preferred terminology (PT)	Palbociclib		Abemaciclib		Ribociclib	
		N	ROR (95% CI)	N	ROR (95% CI)	N	ROR (95% CI)
Cholestasis and jaundice	Hyperbilirubinaemia	—	—	—	—	16	1.91 (1.17, 3.13)
	Jaundice	—	—	20	2.51 (1.62, 3.90)	59	2.44 (1.89, 3.15)
Hepatic and hepatobiliary disorders NEC	Hepatic cyst	—	—	—	—	8	2.78 (1.39, 5.57)
	Hepatic lesion	30	2.06 (1.44, 2.95)	—	—	42	12.13 (8.95, 16.45)
	Hepatic mass	—	—	—	—	26	17.44 (11.83, 25.71)
	Hepatobiliary disease	—	—	—	—	7	15.01 (7.12, 31.68)
	Liver disorder	192	1.28 (1.11, 1.48)	43	3.65 (2.71, 4.93)	107	2.99 (2.47, 3.61)
Hepatic enzymes and function abnormalities	Hepatic function abnormal	—	—	79	8.29 (6.65, 10.35)	—	—
	Hypertransaminasaemia	39	2.58 (1.88, 3.54)	7	5.85 (2.79, 12.29)	14	3.85 (2.28, 6.51)
Hepatic failure and associated disorders	Hepatic failure	—	—	30	3.50 (2.44, 5.00)	52	1.99 (1.52, 2.61)
Hepatic fibrosis and cirrhosis	Hepatic cirrhosis	—	—	11	2.28 (1.26, 4.11)	—	—
Hepatobiliary function diagnostic procedures	Alanine aminotransferase abnormal	—	—	—	—	9	5.82 (3.02, 11.20)
	Alanine aminotransferase increased	—	—	38	2.18 (1.58, 2.99)	196	3.70 (3.22, 4.26)
	Aspartate aminotransferase abnormal	—	—	—	—	4	3.78 (1.42, 10.09)
	Aspartate aminotransferase increased	—	—	36	2.38 (1.71, 3.30)	183	3.99 (3.45, 4.61)
	Bilirubin conjugated increased	—	—	—	—	5	2.47 (1.03, 5.95)
	Blood bilirubin abnormal	—	—	—	—	8	8.25 (4.11, 16.54)
	Blood bilirubin increased	—	—	23	2.95 (1.96, 4.45)	74	3.13 (2.49, 3.93)
	Gamma-glutamyltransferase abnormal	—	—	—	—	4	9.13 (3.41, 24.45)
	Gamma-glutamyltransferase increased	—	—	17	2.58 (1.60, 4.15)	81	4.05 (3.25, 5.04)
	Hepatic enzyme abnormal	39	2.33 (1.70, 3.20)	4	3.03 (1.13, 8.07)	21	5.24 (3.41, 8.05)
	Hepatic enzyme increased	—	—	38	2.21 (1.61, 3.04)	177	3.40 (2.93, 3.94)
	Liver function test abnormal	—	—	—	—	61	2.25 (1.75, 2.90)
	Liver function test decreased	—	—	—	—	3	11.73 (3.76, 36.64)
	Liver function test increased	119	1.91 (1.59, 2.29)	34	6.93 (4.95, 9.70)	119	8.01 (6.69, 9.60)
	Transaminases abnormal	—	—	—	—	3	6.57 (2.11, 20.44)
	Transaminases increased	—	—	12	1.93 (1.09, 3.40)	78	4.13 (3.31, 5.16)
Hepatobiliary neoplasms benign	Haemangioma of liver	—	—	—	—	3	3.25 (1.05, 10.10)
Hepatobiliary neoplasms malignancy unspecified	Hepatic neoplasm	—	—	—	—	21	7.57 (4.93, 11.63)
Hepatobiliary signs and symptoms	Hepatic pain	27	1.81 (1.24, 2.64)	—	—	19	5.31 (3.39, 8.34)
	Hepatomegaly	—	—	—	—	28	3.15 (2.18, 4.57)
Hepatocellular damage and hepatitis NEC	Drug-induced liver injury	—	—	31	4.68 (3.29, 6.66)	34	1.68 (1.20, 2.36)
	Hepatic cytolysis	—	—	12	4.79 (2.72, 8.45)	—	—
	Hepatitis	—	—	—	—	42	1.98 (1.46, 2.68)
	Hepatitis acute	—	—	—	—	12	2.20 (1.25, 3.87)
	Hepatitis toxic	—	—	—	—	9	3.88 (2.01, 7.46)
	Hepatotoxicity	—	—	35	6.08 (4.36, 8.47)	95	5.43 (4.44, 6.65)
	Liver injury	—	—	15	2.98 (1.79, 4.94)	40	2.61 (1.91, 3.56)
Peritoneal and retroperitoneal disorders	Ascites	202	1.94 (1.69, 2.22)	17	2.06 (1.28, 3.32)	104	4.16 (3.43, 5.05)

NEC, not elsewhere classified; ROR, reporting odds ratio; CI, confidence interval; N, number of reports; statistically significant (lower limit of the 95% CI>1 and N>3).

### 3.3 Drug‒hepatic injury–related gene interaction network analysis

**FIGURE 3:**
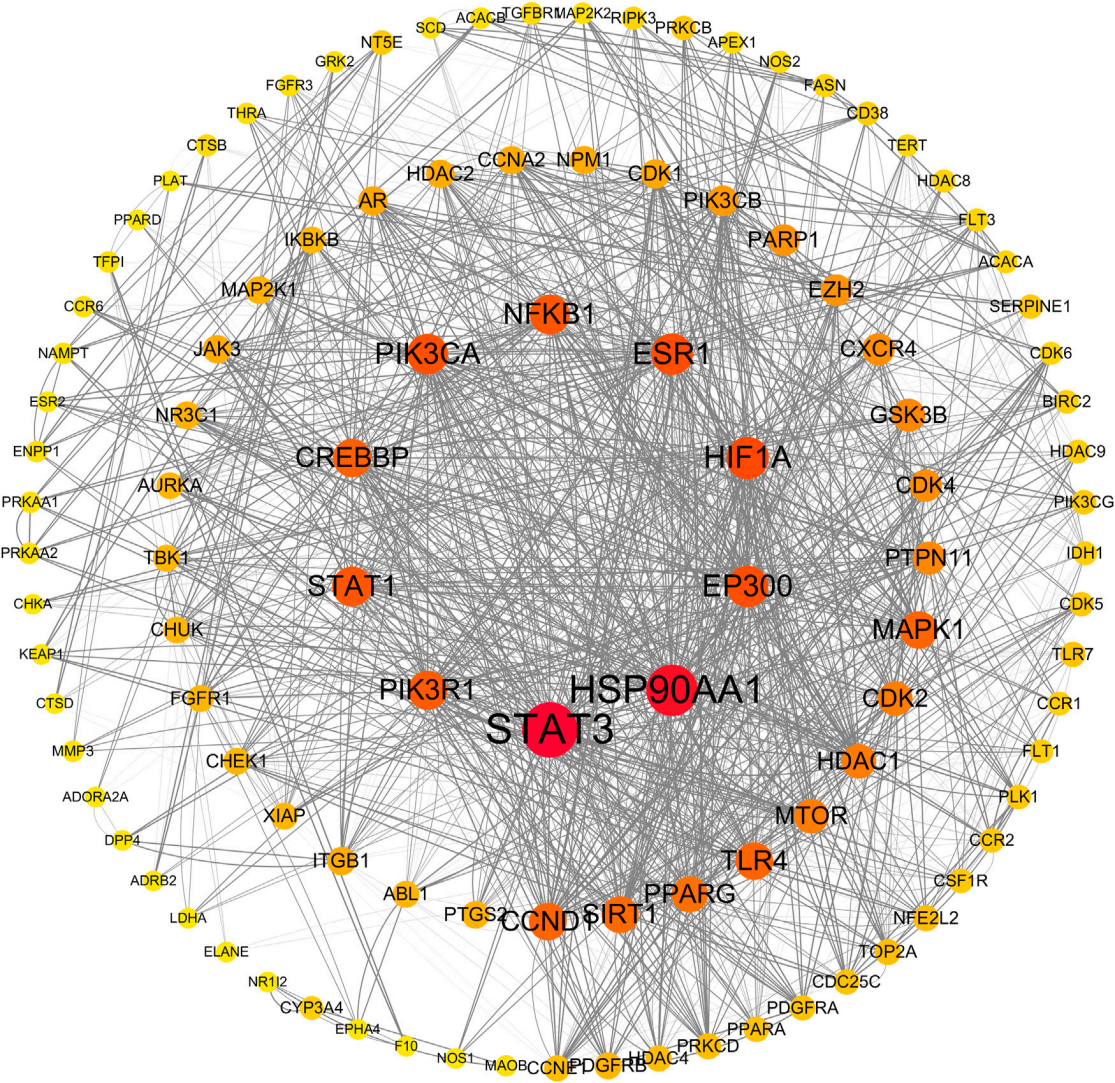
Protein-protein interaction network by Cytoscape. The size and color of nodes in the network represent the degree value, indicating the number of interactions each protein has with other proteins. Larger nodes indicate higher degrees, suggesting greater centrality in biological processes. Edge thickness reflects the magnitude of the combined score, with thicker edges indicating higher combined scores. A higher combined score suggests a stronger likelihood of genuine interactions between proteins, providing insights into the network’s overall connectivity and functional relevance.

After deduplicating the database, we identified 395 target genes associated with abemaciclib, ribociclib, and palbociclib, as well as 2,697 genes linked to liver injury. By intersecting these gene sets, we isolated 189 interactive target genes representing the intersection of CDK4/6 inhibitor targets and genes involved in hepatic injury. The intersecting genes were subjected to protein‒protein interaction (PPI) prediction via the String database (https://string-db.org), facilitating the construction of a protein interaction network using Cytoscape 3.7.2 software. The results are shown in Figure 3. Upon topological analysis, key targets (the centermost circle of nodes) of the interaction were revealed, including STAT3, HSP90AA1, EP300, HIF1A, ESR1, PIK3CA, NFKB1, STAT1, PIK3R1, and CREBBP, revealing their centrality within the network. In addition, we found that CCND1, SIRT1, and PPARG are potential targets for interaction.

**FIGURE 4:**
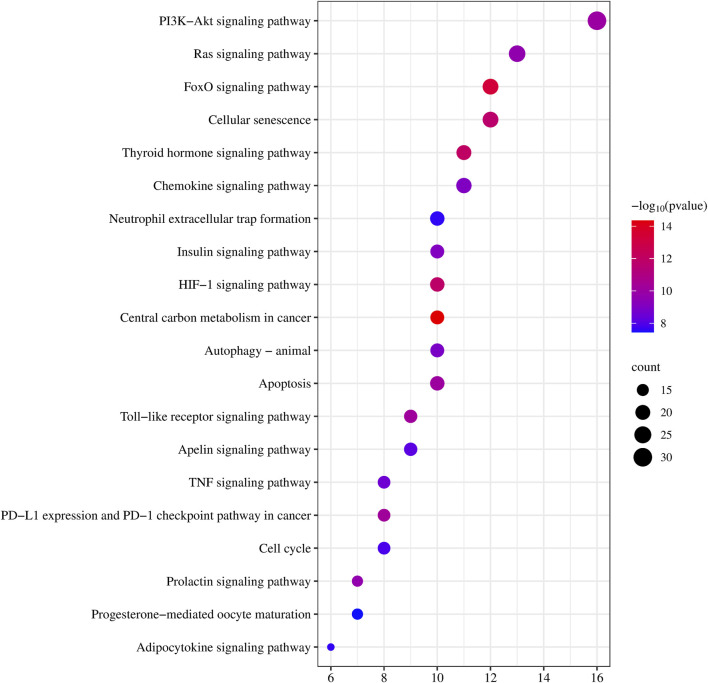
KEGG pathway enrichment analysis. Each bubble represents a specific pathway. The horizontal axis represents the number of genes enriched in each pathway, while the size of the bubbles indicates the extent of enrichment for the corresponding pathway. Color indicates significance, with a gradient from blue to red representing decreasing *p*-values.

To better understand the involvement of CDK4/6 inhibitor-induced liver injury target genes in biological signaling pathways, we conducted KEGG pathway enrichment analysis. We focused on the top 20 pathways for comprehensive mapping, as illustrated in Figure 4. The analysis revealed enrichment of genes interacting with CDK4/6is in various pathways, notably, central carbon metabolism in cancer, the FoxO signaling pathway, insulin resistance, the HIF-1 signaling pathway, cellular senescence, microRNAs in cancer, PD-L1 expression and the PD-1 checkpoint pathway in cancer, the Toll-like receptor signaling pathway, apoptosis, small cell lung cancer, and the PI3K-Akt signaling pathway. These findings strongly suggest the potential association of CDK4/6 inhibitors with the development of liver injury through modulation of these pathways.

## 4 Discussion

With the expansion of CDK4/6is for the treatment of breast cancer, the balance between efficacy and safety has become critical. Our study revealed the safety of ribociclib, abemaciclib and palbociclib, emphasizing the differences in the relative risk of DILI. Ribociclib and abemaciclib demonstrated significant signs of hepatobiliary toxicity, whereas palbociclib appeared relatively safe. Our findings are consistent with previous randomized controlled trials in which hepatobiliary toxicity was more prominent in patients treated with ribociclib and abemaciclib than in controls, making our study the first to comprehensively compare the risk of liver injury with that of these CDK4/6is (Johnston et al., 2020; Onesti and Jerusalem, 2021; Lu et al., 2022).

There is a potential association between hepatic and biliary toxicity and factors such as lipolysis, mitochondrial injury, metabolism and hepatic transporters (Gu and Manautou, 2012). The high lipophilicity of abemaciclib may be the factor responsible for its association with more hepatic adverse effects than palbociclib (Chen et al., 2016). Ribociclib inhibits hepatic transporters, such as bile salt efflux pumps (BSEPs), and the basal outflow system and may therefore induce additional DILI signals (Rana et al., 2019; Jetter and Kullak-Ublick, 2020). Moreover, palbociclib lacks BSEP inhibition and mitochondrial toxicity and therefore has a relatively low hepatotoxicity signal (Rana et al., 2019; Raschi and De Ponti, 2019).

Notably, our study revealed previously undetected hepatotoxic adverse events associated with CDK4/6is. While clinical trials have focused on laboratory-sensitive AEs, spontaneous reporting data have provided essential real-world insights, emphasizing the significance of vigilant pharmacovigilance for identifying rare adverse reactions (FDA, 2009; Lucas et al., 2022). AE analysis of palbociclib revealed liver-related signals (ascites, liver disorders, increased liver function), consistent with increased risk in new and long-term users (Beachler et al., 2021; Finn et al., 2021). However, palbociclib labels lack specific liver risk warnings, and no recommended liver function tests may pose safety risks during prolonged use. Healthcare providers should consider regular liver function monitoring for long-term palbociclib patients.

The integration of pharmacogenetic network analysis revealed important insights into the underlying molecular mechanisms involved in CDK4/6is-induced DILI. The constructed protein interaction network highlighted STAT3, HSP90AA1 and EP300 as key players, suggesting that they play important roles in mediating the interaction between CDK4/6is and liver injury pathways (Gao et al., 2012; Jiao et al., 2023). The association of STAT3 with hepatic inflammation and fibrosis is particularly noteworthy, providing a further avenue for exploring the effects of CDK4/6is on these processes. It is known that activating hepatic STAT3 can prevent inflammation by inhibiting the proinflammatory signaling of STAT1 (Gao et al., 2012). However, it may also promote inflammation by inducing hepatocyte-derived acute-phase proteins. In terms of fibrosis, inhibiting components of hepatic STAT3 activation has shown promise in attenuating hepatic fibrosis, suggesting a complex interplay in liver pathophysiology (Zhao et al., 2021; Lee and Hoe, 2023).

HSP90AA1, a molecular chaperone involved in protein folding and stabilization, is potentially implicated in alcoholic hepatitis and cirrhosis (Choudhury et al., 2020; Costa et al., 2020). EP300, a histone acetyltransferase, has been linked to multiorgan fibrosis through the TGFβ pathway, suggesting epigenetic regulation of fibrogenesis and progression (Rubio et al., 2023). These findings provide avenues for future studies of the precise mechanisms by which CDK4/6 inhibitors influence these key molecular players in liver pathophysiology.

SIRT1 (Sirtuin 1) is a member of the Sirtuin family and acts as a nicotinamide adenine dinucleotide (NAD)-dependent deacetylase. It plays an important role in various physiological processes, including metabolism and aging (Rahman and Islam, 2011; Martins, 2016; Martins, 2017a; Martins, 2017b). Our investigation revealed that SIRT1 could be one of the proteins that interact with CDK4/6 inhibitors leading to liver injury. Given its integral role in liver function, prior studies have linked the downregulation of SIRT1 to the onset and progression of non-alcoholic fatty liver disease (NAFLD) (Colak et al., 2011; Martins, 2017c). Consequently, we posited that hepatic SIRT1 activity might be attenuated by CDK4/6 inhibitors, potentially precipitating hepatotoxicity. However, no empirical study has yet confirmed the impact of CDK4/6 inhibitors on SIRT1 activity. As a result, further empirical investigations are required to validate this assumption.

Understanding the differential risks and underlying mechanisms of CDK4/6 inhibitor-induced liver injury has pivotal clinical implications for treatment decisions and drug development. Our findings pave the way for targeted interventions, biomarker discoveries, and personalized treatment strategies aimed at mitigating hepatotoxicity risks associated with CDK4/6 inhibitors.

Despite the advantages of utilizing the FAERS database and data mining techniques in our study, there are inherent limitations (self-reporting nature of the database, incomplete data and bias) (Alomar, 2014). Second, the database included only reported cases of AEs, and the denominator for the incidence of AEs was unknown. Finally, FAERS-based disproportionality analyses cannot indicate causality or quantify risk; rather, they can only show signal strength and statistical associations without pharmacological mechanism studies. Although our study investigated the potential mechanisms of liver injury caused by CDK4/6is through the examination of drug–gene networks, further research is necessary to validate and expand upon our findings.

## 5 Conclusion

In conclusion, our study sheds light on the differential risk of drug-induced liver injury among CDK4/6 inhibitors, unravels potential mechanistic insights through drug–gene network analysis, and highlights central molecular targets. These findings hold significant clinical implications and pave the way for further investigations, potentially guiding the development of safer and more effective therapies for breast cancer patients.

## Data Availability

The original contributions presented in the study are included in the article/supplementary material, further inquiries can be directed to the corresponding authors.
